# Supplementary material for: Value of dynamic clinical and biomarker data for mortality risk prediction in COVID-19: a multicentre retrospective cohort study
Source: BMJ Open. 2020 Sep 23;10(9):e041983. doi: 10.1136/bmjopen-2020-041983 (PMC7513423; doi:10.1136/bmjopen-2020-041983)
Supplement: Supplementary data [file bmjopen-2020-041983supp001.pdf]

	Day 1	Day 2	Day 3	Day 4	Day 5
White Cell count x10^9/L	7·5 (4·2)	7·1 (4·3)	7·5 (4·3)	7·1 (4·7)	7·6 (4·6)
Lymphocytes x10^9/L	0·8 (0·6)	0·8 (0·5)	0·8 (0·5)	0·8 (0·6)	0·8 (0·5)
Neutrophils x10^9/L	6·1 (4·6)	5·7 (4·2)	5·6 (4·2)	5·6 (4·4)	6·4 (4·2)
Platelets x10^9/L	202 (121)	209 (125)	235 (131)	262 (161)	265 (164)
Bilirubin mg/dL	10 (8)	9 (7)	11 (7)	10(5)	11 (6)
Urea mmol/L	8 (6)	7(6)	7(6)	7(6)	8 (6)
Creatinine µmol/L	90 (56)	80(51)	74 (45)	75(40)	74 (52)
CRP mg/ml	98 (123)	115 (98)	122 (121)	121 (125)	117 (146)

Supplementary Table: Values of biomarkers at each day. Data is presented as median (interquartile range).
