# Supplementary material for: Value of dynamic clinical and biomarker data for mortality risk prediction in COVID-19: a multicentre retrospective cohort study
Source: BMJ Open. 2020 Sep 23;10(9):e041983. doi: 10.1136/bmjopen-2020-041983 (PMC7513423; doi:10.1136/bmjopen-2020-041983)
Supplement: Author's manuscript [file bmjopen-2020-041983.draft_revisions.pdf]

BMJ Open is committed to open peer review. As part of this commitment we make the peer review history of every article we publish publicly available.

When an article is published we post the peer reviewers' comments and the authors' responses online. We also post the versions of the paper that were used during peer review. These are the versions that the peer review comments apply to.

The versions of the paper that follow are the versions that were submitted during the peer review process. They are not the versions of record or the final published versions. They should not be cited or distributed as the published version of this manuscript.

BMJ Open is an open access journal and the full, final, typeset and author-corrected version of record of the manuscript is available on our site with no access controls, subscription charges or pay-per-view fees (http://bmjopen.bmj.com).

If you have any questions on BMJ Open's open peer review process please email info.bmjopen@bmj.com

Dynamic clinical and biomarker data for mortality risk prediction in COVID-19

Journal:	*BMJ Open*
Manuscript ID	bmjopen-2020-041983
Article Type:	Original research
Date Submitted by the Author:	23-Jun-2020

Complete List of Authors:	Berzuini, Carlo; University of Manchester, Biostatistics<br>Hannan, Cathal; Salford Royal Hospitals NHS Trust, Manchester Centre for Clinical Neurosciences<br>King, Andrew; Salford Royal Hospitals NHS Trust, Manchester Centre for Clinical Neurosciences<br>Vail, Andy; University of Manchester, Biostatistics<br>O'Leary, Claire; The University of Manchester, Division of Neuroscience and Experimental Psychology<br>Brough, David; The University of Manchester, Division of Neuroscience and Experimental Psychology<br>Galea, James; Cardiff and Vale University Health Board<br>Ogungbenro, Kayode; The University of Manchester, Pharmacy and Optometry<br>Wright, Megan; Salford Royal Hospitals NHS Trust, Manchester Centre for Clinical Neurosciences<br>Pathmanaban, Omar; Salford Royal Hospitals NHS Trust, Manchester Centre for Clinical Neurosciences<br>Hulme, Sharon; The University of Manchester, Division of Neuroscience and Experimental Psychology<br>Allan, Stuart; The University of Manchester, Division of Neuroscience and Experimental Psychology<br>Bernardinelli, Luisa; University of Pavia<br>Patel, Hiren; Salford Royal Hospitals NHS Trust, Manchester Centre for Clinical Neurosciences

Keywords:	STATISTICS & RESEARCH METHODS, Respiratory infections < THORACIC MEDICINE, INTENSIVE & CRITICAL CARE

SCHOLARONE™
Manuscripts

I, the Submitting Author has the right to grant and does grant on behalf of all authors of the Work (as defined in the below author licence), an exclusive licence and/or a non-exclusive licence for contributions from authors who are: i) UK Crown employees; ii) where BMJ has agreed a CC-BY licence shall apply, and/or iii) in accordance with the terms applicable for US Federal Government officers or employees acting as part of their official duties; on a worldwide, perpetual, irrevocable, royalty-free basis to BMJ Publishing Group Ltd ("BMJ") its licensees and where the relevant Journal is co-owned by BMJ to the co-owners of the Journal, to publish the Work in this journal and any other BMJ products and to exploit all rights, as set out in our licence.

The Submitting Author accepts and understands that any supply made under these terms is made by BMJ to the Submitting Author unless you are acting as an employee on behalf of your employer or a postgraduate student of an affiliated institution which is paying any applicable article publishing charge ("APC") for Open Access articles. Where the Submitting Author wishes to make the Work available on an Open Access basis (and intends to pay the relevant APC), the terms of reuse of such Open Access shall be governed by a Creative Commons licence – details of these licences and which Creative Commons licence will apply to this Work are set out in our licence referred to above.

Other than as permitted in any relevant BMJ Author's Self Archiving Policies, I confirm this Work has not been accepted for publication elsewhere, is not being considered for publication elsewhere and does not duplicate material already published. I confirm all authors consent to publication of this Work and authorise the granting of this licence.

For peer review only

Dynamic clinical and biomarker data for mortality risk prediction in COVID-19

Carlo Berzuini PhD, Cathal Hannan MRCS, Andrew Thomas King FRCS(SN), Andy Vail MSc, Claire O'Leary PhD, David Brough PhD, James Galea PhD, Kayode Ogungbenro PhD, Megan Wright BSc, Omar Pathmanaban PhD, Sharon Hulme BSc, Stuart Allan PhD, Luisa Bernardinelli PhD, Hiren Patel PhD *.

Carlo Berzuini (carlo.berzuini@manchester.ac.uk). Professor of Biostatistics, Centre for Biostatistics, University of Manchester, Oxford Road, Manchester, M13 3PL, UK

Cathal John Hannan (cathalhannan@icloud.com). Neurosurgery Research Registrar, Manchester Centre for Clinical Neurosciences, Stott Lane, Manchester, M6 8HD, UK

Andrew Thomas King (Andrew.king@manchester.ac.uk). Professor of Neurosurgery, Manchester Centre for Clinical Neurosciences, Stott Lane, Manchester, M6 8HD, UK

Andy Vail (andy.vail@manchester.ac.uk). Professor of Biostatistics and Centre Lead, Centre for Biostatistics, University of Manchester, Oxford Road, Manchester, M13 3PL, UK

Claire O'Leary (Claire.oleary@manchester.ac.uk). Post-doctoral research associate, Division of Neuroscience and Experimental Psychology, University of Manchester, Oxford Road, Manchester, M13 3PL, UK

David Brough (David.brough@manchester.ac.uk). Professor of Neuroscience, Division of Neuroscience and Experimental Psychology, University of Manchester, Oxford Road, Manchester, M13 3PL, UK

James Galea (james.galea@manchester.ac.uk). Consultant Neurosurgeon, Cardiff and Vale University Health Board, Heath Park, Cardiff, CF14 4XW, UK

For peer review only - http://bmjopen.bmj.com/site/about/guidelines.xhtml

Kayode Ogungbenro (Kayode.Ogungbenro@manchester.ac.uk). Lecturer in Cancer Pharmocometrics, Division of Pharmacy and Optometry, University of Manchester, Oxford Road, Manchester, M13 3PL, UK

Megan Wright (megan.wright@manchester.ac.uk). Research Manager, Manchester Centre for Clinical Neurosciences, Stott Lane, Manchester, M6 8HD, UK

Omar Pathmanaban (omar.pathmanaban@manchester.ac.uk). Consultant Neurosurgeon, Manchester Centre for Clinical Neurosciences, Stott Lane, Manchester, M6 8HD, UK

Sharon Hulme (sharon.hulme@manchester.ac.uk). Research Manager, Division of Cardiovascular Sciences, University of Manchester, Oxford Road, Manchester, M13 3PL, UK

Stuart Allan (stuart.allan@manchester.ac.uk). Professor of Neuroscience, Division of Neuroscience and Experimental Psychology, University of Manchester, Oxford Road, Manchester, M13 3PL, UK

Luisa Bernardinelli (luiber04@unipv.it). Professor of Biostatistics, University of Pavia, Via Bassi, 21 - 27100 Pavia, Italy

Hiren Patel (hiren.patel@srft.nhs.uk). Consultant Neurosurgeon, Manchester Centre for Clinical Neurosciences, Stott Lane, Manchester, M6 8HD, UK

For peer review only

Contributor and Guarantor Information

All authors were involved in the concept and design of the study which was led by HP and AV. HP, CH, MW acquired the data, and CB, LB, AV, HP, CH analysed and interpreted the data. CB, DB, SA, OP, JG, AV, CH, HP drafted the manuscript. All authors were involved in revising the manuscript and contributed to the final draft. The corresponding author attests that all listed authors meet authorship criteria and that no others meeting the criteria have been omitted. HP acts as guarantor for the study.

Copyright

The Corresponding Author has the right to grant on behalf of all authors and does grant on behalf of all authors, a worldwide licence to the Publishers and its licensees in perpetuity, in all forms, formats and media (whether known now or created in the future), to i) publish, reproduce, distribute, display and store the Contribution, ii) translate the Contribution into other languages, create adaptations, reprints, include within collections and create summaries, extracts and/or, abstracts of the Contribution, iii) create any other derivative work(s) based on the Contribution, iv) to exploit all subsidiary rights in the Contribution, v) the inclusion of electronic links from the Contribution to third party material where-ever it may be located; and, vi) licence any third party to do any or all of the above."

Competing Interests Declaration

Swedish Orphan Biovitrum have provided investigational medicinal product for public-funded, peer-reviewed trials on which AK, AV, JG, HP, and SH are coinvestigators. The other authors declare no competing interests.

For peer review only

*Corresponding author: Manchester Centre for Clinical Neurosciences, Salford Royal NHS Foundation Trust, Manchester Academic Health Science Centre, Manchester, M6 8HD, UK.

Telephone:01612065059. Email: Hiren.Patel@srft.nhs.uk

Word Count 1357

Number of figures: 4

Number of tables: 2

For peer review only - http://bmjopen.bmj.com/site/about/guidelines.xhtml

Abstract

Objectives

Being able to predict which COVID-19 patients are going to deteriorate is important to help identify patients for clinical and research practice. Clinical prediction models play a critical role in this process, but current models are of limited value because they are typically restricted to baseline predictors and don't always use state of the art methods. We sought to explore the benefits of incorporating dynamic changes in routinely measured biomarkers, non-linear effects and applying 'state of the art' statistical methods in the development of a prognostic model to predict death in hospitalised COVID-19 patients.

Design

Data were analysed from COVID-19 admissions to three hospital sites. Exploratory data analysis included a graphical approach to partial correlations. Dynamic biomarkers were considered up to five days following admission rather than depending solely on baseline or single time-point data. Marked departures from linear effects of covariates were identified by employing smoothing splines within a generalised additive modelling framework.

Setting

3 secondary and tertiary level centres in Greater Manchester

Participants

392 hospitalised patients with a diagnosis of COVID-19

Results

392 patients with a COVID-19 diagnosis were identified. Area under the receiver operating characteristic (ROC) curve increased from 0.73 using admission data alone to 0.75 when also considering results of baseline blood samples and to 0.83 when considering dynamic values of routinely collected markers. There was clear non-linearity in the association of age with patient outcome.

Conclusions

This study shows that clinical prediction models to predict death in hospitalised COVID-19 patients can be improved by taking into account both non-linear effects in covariates such as age and dynamic changes in values of biomarkers.

For peer review only

Strengths and Limitations.

- Using contemporary statistical methods, and by incorporating routinely available blood tests performed over the first 5 days of hospital admission we have shown the importance of using dynamic blood biomarker data to enhance patient-level prediction of COIVD-19 progression.
- This approach should inform how future clinical prediction models are generated.
- We did not have sufficient data to construct definitive prediction models.
- More sophisticated exploitation of biomarker trajectories through, for example, approaches based on random effects models of biomarker evolution or 'conditional on outcome' models of biomarker evolution, could make clinical predictions models better still.

For peer review only

Introduction

Most patients with severe acute respiratory syndrome coronavirus 2 (SARS-Cov-2) experience mild symptoms. Some patients however experience significant symptoms requiring hospitalisation. The pandemic nature of the covid-19 outbreak has meant that hospital services and capacity can be overwhelmed.[1] A tool to predict which patients are likely to deteriorate or need intensive care would help clinicians, hospital managers and researchers make better decisions.

Several such models are reported for COVID-19 patients but have been criticised for risk of bias using the PROBAST (prediction model risk of bias assessment tool) criteria.[2] We have further concerns regarding the statistical tools used to develop models. First, current models typically only consider patient characteristics available at baseline and do not consider that COVID-19 patients' presentation and in hospital course is variable. Secondly, models routinely seek only linear effects of potential predictors on the outcome of interest although these are not always clinically plausible.

We sought here to explore the benefits of incorporating dynamic changes in routinely measured biomarkers, non-linear effects and applying 'state of the art' statistical methods in the development of a prognostic model to predict death in hospitalised COVID-19 patients.

Methods

Study Population

Admissions with confirmed COVID-19 (according to World Health Organisation guidance) at three hospitals in the Northern Care Alliance (Greater Manchester, UK) between 11th March and 17th April 2020 with a minimum of a three week follow-up were studied.[3]

Data Collection

Necessary approvals were obtained from the local Research and Innovation department. Research nurses abstracted data from the electronic patient records based on the International Severe Acute Respiratory and emerging Infection Consortium (ISARIC) data collection tool but modified for use with this study.[4] The ISARIC study data were supplemented from electronic patient records with results of blood analyses performed as part of routine clinical care. The date of diagnosis was considered Day 1.

For peer review only - http://bmjopen.bmj.com/site/about/guidelines.xhtml

Data analysis

All data were subjected to range checks and validated for internal consistency and missing items then anonymised prior to transfer.

Selection of biomarkers

The initial list of potential markers was determined through review of the literature and availability within routinely collected data. Candidate variables were further screened using a graphical representation of the partial correlation structure stratified by survival status.[5] Routine bloods were typically analysed on alternate days. The assumption that the unrecorded values were missing at random was corroborated by inspection of joint bivariate plots of complete and incomplete observations made on each particular marker on consecutive days.[6] Then, each missing value of a marker was imputed by iterative sampling from its conditional predictive distribution given its past values, using R package MICE. [6 7]

Modelling

A binary logistic model for the all-cause mortality outcome using only clinical features at presentation was fitted initially. For each of the first five days following admission, additional potential predictors from the routinely measured biomarkers available by that day were then selected resulting in a sequence of day specific mortality prediction models. We subsequently fitted each model within a generalised additive modelling (GAM) framework involving smoothing splines to detect marked departures from linearity for continuous predictors and undertook data transformations (e.g. log transformation of concentrations) as indicated.[8] A standard logistic version of the model was then fitted. We used the Akaike Information Criterion (AIC) to choose between logistic models and assessed predictive performance using the area under a ten-fold, cross-validated Receiver Operating Characteristic (ROC) curve.

Results

A total of 392 patients with a COVID-19 diagnosis were admitted during the study period. Table 1 provides a summary of their demographic and clinical features including medical history. Blood samples were typically requested every other day following admission (supplementary Table).

For peer review only - http://bmjopen.bmj.com/site/about/guidelines.xhtml

For an informal analysis of biomarker relationships we analysed partial correlations between seven potential inflammatory markers in each of the two survival groups (Fig 1). Amongst survivors, the anticipated correlations between lymphocytes (Lym) and neutrophils (Neu, via white cell count) and between creatinine (Cre) and urea (Ure) were present, but these correlations were found to be significantly lower among the decedents, suggesting the possible presence of differences in the neutrophil/lymphocyte and urea/creatinine ratios between the two outcome groups.

Inclusion of admission biomarker data did not improve the predictive value of the model over clinical data alone. Incorporation of post admission dynamic biomarker data did however increase the discriminative ability of the model (Fig 2). Estimates from the best fitting model at Day 5 (Table 2) show strongly statistically significant term(s) reflecting post-baseline biomarker changes that that can be readily visualised (Fig 3). In addition to age and disease severity, the most recent neutrophil:lymphocyte ratio and the two most recent (and therefore recent change in) urea:creatinine ratios were generally predictive. There was a marked non-linearity in the effect of age (Fig 4).

Discussion

These results suggest that using dynamic data is better than using baseline initial presentation data to predict death in COVID-19 patients. Even with a local dataset of just 392 COVID-19 admissions we were able to identify clear benefit from exploiting dynamic biomarker data and marked non-linearity in the effects of commonly used factors to predict outcomes. Our findings should be taken as indicative of the benefit of applying more recent developments in statistical methodology than are commonly found in the clinical literature. Identification and validation of anything approaching a definitive predictive model would require substantially larger sample sizes.[9]

Neither the non-linear effect of age after allowance for other factors nor the particular biomarkers identified within this dataset are surprising. Others have also observed associations of mortality with age, and clinical and biochemical markers of disease severity (e.g. neutrophil/lymphocyte ratio).[10-12] Similarly, renal injury has also been shown to be common in COVID-19 patients and is associated with a worse outcome.[13] The reason for this is not clear. There is emerging evidence that SARS-CoV-2 infection can directly harm the kidneys. The worsening urea/creatinine ratio observed in our data set may also reflect either the therapeutic effects of fluid restriction to treat severe ARDS or evidence of multiorgan

For peer review only - http://bmjopen.bmj.com/site/about/guidelines.xhtml

dysfunction.[11 14] Regardless of the cause, the impact of the urea/creatinine level on death was not evident at presentation but became a significant predictor of death in our model over time. This illustrates the benefit of taking into account improvements and deterioration in daily blood test results as well as initial presentation factors when calculating the probability of death. Improving the accuracy of prediction models using this approach is likely to be successful in informing clinical decision making, resource planning and communication with patients and relatives.

We acknowledge that our study has some limitations. We would have liked to consider other outcomes including ARDS and ICU admission, but a consistent diagnosis and dates were not routinely available. Although we have undertaken internal cross-validation to ensure unbiased comparison of ROC curves we have not considered calibration. We do not wish to make any claim for the value of our current models at each day based on the small sample size available to us locally. With three hospital sites contributing during the first wave of the pandemic we do not have sufficient data to construct definitive prediction models. More sophisticated exploitation of biomarker trajectories through, for example, approaches based on random effects models of biomarker evolution or 'conditional on outcome' models of biomarker evolution, would also require more data and be expected to add further insights.[15-17]

Clinical prediction models are important and can help in clinical decision making, resource allocation and optimal selection of trial participants for investigational treatments. In the setting of an infectious disease pandemic- affecting all geographic and socioeconomic groups – using routinely available and performed blood tests to inform prediction models has obvious advantages over less widely available, but perhaps more specific, biomarkers of disease severity. Until investigators incorporate such data in participant selection it is unlikely that future trials will be able to accurately target those patients most likely to benefit from therapies such as immunomodulation. Overall benefits will be 'diluted' and potentially reversed by inclusion of participants who have nothing to gain and, in theory, may be harmed by restriction of a healthy inflammatory response.[18] The consequence of poorly considered eligibility criteria may therefore be to erroneously dismiss therapies that could benefit those at highest risk from COVID-19.

Data sharing statement.

Our data would be made available to reasonable requests.

For peer review only

Patient and Public involvement.

There was no involvement of patients or public involvement in the design or delivery of this study. This was because of the acute nature and fast moving pace of the disease studied and because access to patients and the public was limited at this time.

References

1. Emanuel EJ, Persad G, Upshur R, et al. Fair Allocation of Scarce Medical Resources in the Time of Covid-19. *New England Journal of Medicine* 2020;382(21):2049-55. doi: 10.1056/NEJMsb2005114
2. Wynants L, Van Calster B, Collins GS, et al. Prediction models for diagnosis and prognosis of covid-19: systematic review and critical appraisal. *BMJ* 2020;369:m1328. doi: 10.1136/bmj.m1328
3. WHO. Laboratory testing for coronavirus disease (COVID-19) in suspected human cases. 2020
4. International Severe Acute Respiratory and emerging Infection Consortium. URL: https://isaric.tghn.org Accessed 28/05/2020.
5. Friedman J, Hastie T, Tibshirani R. Sparse inverse covariance estimation with the graphical lasso. *Biostatistics* 2007;9(3):432-41. doi: 10.1093/biostatistics/kxm045
6. RUBIN DB. Inference and missing data. *Biometrika* 1976;63(3):581-92. doi: 10.1093/biomet/63.3.581
7. Azur MJ, Stuart EA, Frangakis C, et al. Multiple imputation by chained equations: what is it and how does it work? *Int J Methods Psychiatr Res* 2011;20(1):40-9. doi: 10.1002/mpr.329 [published Online First: 2011/04/19]
8. Hastie T, Tibshirani R. Generalized additive models. 2017
9. Wynants L, Van Calster B, Bonten MMJ, et al. Prediction models for diagnosis and prognosis of covid-19 infection: systematic review and critical appraisal. *BMJ* 2020;369:m1328. doi: 10.1136/bmj.m1328
10. Huang C, Wang Y, Li X, et al. Clinical features of patients infected with 2019 novel coronavirus in Wuhan, China. *The Lancet* 2020;395(10223):497-506. doi: 10.1016/S0140-6736(20)30183-5
11. Zhou F, Yu T, Du R, et al. Clinical course and risk factors for mortality of adult inpatients with COVID-19 in Wuhan, China: a retrospective cohort study. *The Lancet* 2020;395(10229):1054-62. doi: 10.1016/S0140-6736(20)30566-3
12. Ruan Q, Yang K, Wang W, et al. Clinical predictors of mortality due to COVID-19 based on an analysis of data of 150 patients from Wuhan, China. *Intensive Care Medicine* 2020;46(5):846-48. doi: 10.1007/s00134-020-05991-x
13. Cheng Y, Luo R, Wang K, et al. Kidney disease is associated with in-hospital death of patients with COVID-19. *Kidney Int* 2020;97(5):829-38. doi: 10.1016/j.kint.2020.03.005 [published Online First: 2020/04/06]
14. Su H, Yang M, Wan C, et al. Renal histopathological analysis of 26 postmortem findings of patients with COVID-19 in China. *Kidney International* doi: 10.1016/j.kint.2020.04.003
15. Berzuini C, Larizza C. A unified approach for modeling longitudinal and failure time data, with application in medical monitoring. *IEEE Transactions on Pattern Analysis and Machine Intelligence* 1996;18(2):109-23.
16. Albert PS. A linear mixed model for predicting a binary event from longitudinal data under random effects misspecification. *Stat Med* 2012;31(2):143-54. doi: 10.1002/sim.4405
17. Liu D, Albert PS. Combination of longitudinal biomarkers in predicting binary events. *Biostatistics* 2014;15(4):706-18. doi: 10.1093/biostatistics/kxu020 [published Online First: 2014/05/17]
18. King A, Vail A, O'Leary C, et al. Anakinra in COVID-19: important considerations for clinical trials. *The Lancet Rheumatology* doi: 10.1016/S2665-9913(20)30160-0

For peer review only - http://bmjopen.bmj.com/site/about/guidelines.xhtml

Acknowledgments

The Northern Care Alliance research delivery team who collected the data for the patients, and the Salford Royal business analysis team who helped in providing access to the daily blood and clinical observation results. This report was also made possible by the provision of the data collection tool through the efforts and expertise of the ISARIC Team (www.isaric.tghn.org).

Role of funding source

AK, AV, JG, HP, and SH are funded by the National Institute for Health Research Efficacy and Mechanism Evaluation Programme Ref: 14/209/07. DB is funded by MRC grant MR/T016515/1.

For peer review only

Figure Legends:

Figure 1. Partial correlations between biomarkers. Nodes represent average marker levels day 2 to 5 and edges represent partial correlations, as calculated from the survivors (left) and from the decedents (right). Broader lines indicate stronger relationships. Blr Bilirubin, CRP- C-Reactive Protein, Crt-Creatinine, Lym-Lymphocytes, Ntr-Neutrophils, WCC- White Cell Count, Ure-Urea.

Figure 2. ROC curves for three models: solid line indicates model considering only clinical factors at baseline (Area under ROC curve = 0.73); finely dotted line indicates model extended to consider also biomarker data from baseline sample (Area under ROC curve = 0.75); top line indicates model at 5 days extended to consider dynamic changes in biomarker data (Area under ROC curve = 0.83). Note that models are not nested

Figure 3. Violin plots showing distribution at each day of admission, stratified by survival status, for biomarkers identified by statistical modelling. Panel A: log transformed neutrophil ($x10^{\wedge 9}$/L):lymphocyte ($x10^{\wedge 9}$/L) ratio. Panel B: log transformed urea (mmol/L):creatinine (µmol/L) ratio. Survivors (white) on left and decedents (shaded) on right.

Figure 4. Spline plot demonstrating marked non-linearity in relationship between age and outcome after adjustment for other factors included in the final model.

For peer review only

Table 1. Demographic, clinical and medical history factors considered at baseline.

	Overall dataset
Number of patients	392
Age, median (IQR)	71(22)
Gender Male: Female ratio	65:35
Median time to hospitalisation following disease onset (IQR) days	5(8)
Initial symptoms (%)	
Fever	223 (57%)
Cough	240(61%)
Dyspnoea	245 (65%)
Fatigue	127 (37%)
Muscle ache	53 (16%)
Co-morbidities	
Cardiovascular disease	108 (28%)
Chronic Respiratory disease (inc asthma)	110 (28%)
Chronic Renal disease	45 (12%)
Chronic Liver disease	14 (2%)
Obesity	34 (10%)
Diabetes	95 (24%)
Dementia	49 (13%)
Current smoker	24 (7%)
Presenting clinical features	
Requirement for supplemental O_2	125(37%)
Oxygen Saturation < 90	59 (17%)
Respiratory rate>24	109 (30%)
Temperature ≥38°C	168 (45%)
MAP≤70mmHg	30 (8%)
Outcomes	
Acute Respiratory Distress Syndrome	47 (17%)
Non-Invasive Ventilation	25 (9%)
Need for ICU care	31 (12%)
Invasive Ventilation	14 (5%)
Death	110 (27%)

For peer review only

Table 2. Estimated coefficients (Est) with their standard error (se) and p-value. Note that different variables are selected at different days so that models are not nested. Neut/Lymp- Neurotrophil/Lymphocyte, Creat- Creatinine, D- Day, O_2- Oxygen. * The addition of biomarker data on day 2 did not contribute any additional predictive power of that obtained at Day 1.

	Clinical data alone Day 1			Clinical data + Day 1* biomarker data			Day 3			Day 4			Day 5		
**Predictor**	**Est**	**se**	***p***	**Est**	**se**	***p***	**Est**	**se**	***p***	**Est**	**se**	***p***	**Est**	**se**	***p***
**Intercept**	0·31	1·36	0·8	-4·36	1·35	0·001	0·67	1·97	0·73	0·005	1·86	0·99	-0·20	1·68	0·9
**log Neut/Lymp D1**				0·28	0·16	0·08									
**log Neut/Lymp D3**							0·41	0·19	0·03						
**log Neut/Lymp D4**										0·48	0·2	0·02			
**log Neut/Lymp D5**													0·52	0·21	0·01
**log Urea/Creat D2**							-4·22	1·24	0·0007						
**log Urea/Creat D3**							5·13	1·30	0·0001						
**log Urea/Creat D4**										1·08	0·35	0·002	-4·97	1·72	0·0003
**log Urea/Creat D5**													6·32	1·77	0·0004
**Age (Years)**	0·13	0·026	<0·0001	0·073	0·012	<0·0001	0·069	0·013	<0·0001	0·071	0·012	<0·0001	0·066	0·012	<0·0001
**O_2 Saturation**	-0·03	0·013	0·05	-0·03	0·012	0·01	-0·03	0·013	0·03	-0·03	0·012	0·02			
**Respiratory Rate**	0·05	0·022	0·02	0·085	0·022	0·0001	0·08	0·023	0·0003	0·087	0·022	0·0001	0·09	0·022	0·0001
**Smoking**	0·44	0·29	0·1	0·7	0·267	0·01	0·8	0·27	0·004	0·71	0·27	0·008	0·76	0·28	0·006

For peer review only

For peer review only - http://bmjopen.bmj.com/site/about/guidelines.xhtml

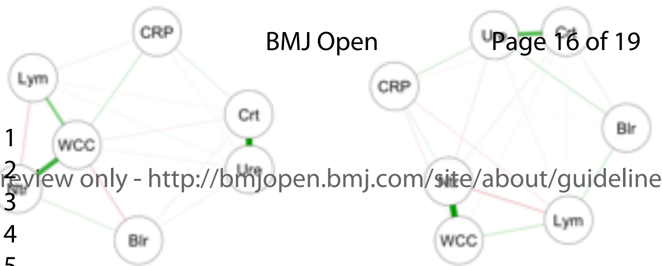

er review only - http://bmjopen.bmj.com/site/about/guideline

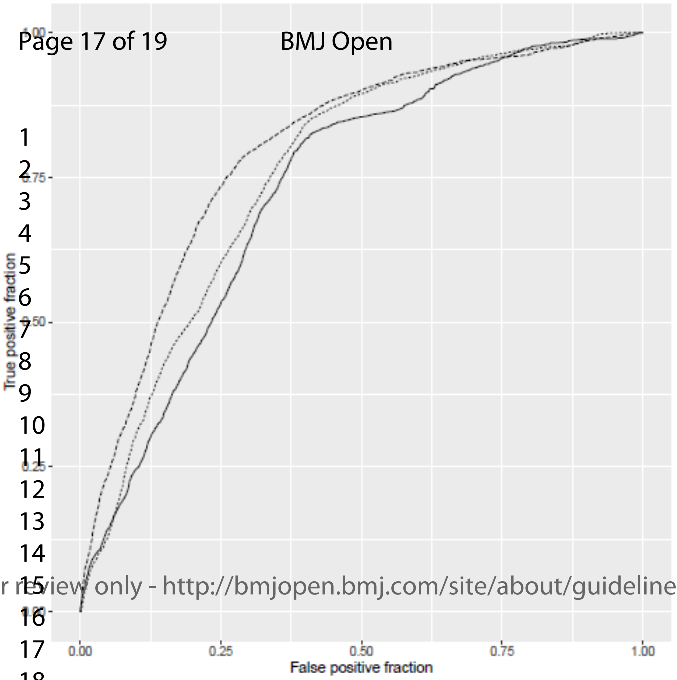

er review only - http://bmjopen.bmj.com/site/about/guideline

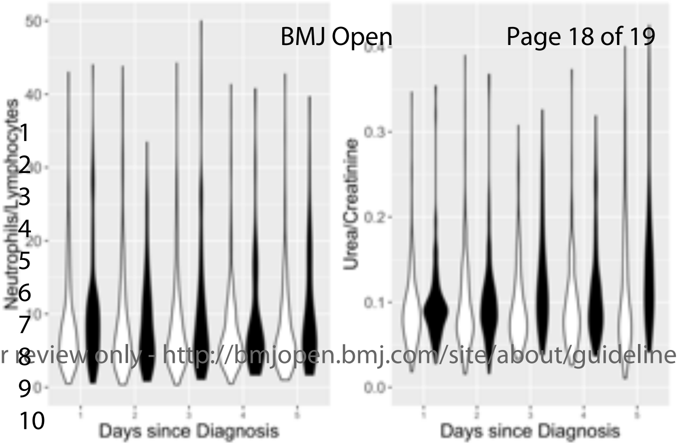

er review only - http://bmjopen.bmj.com/site/about/guideline

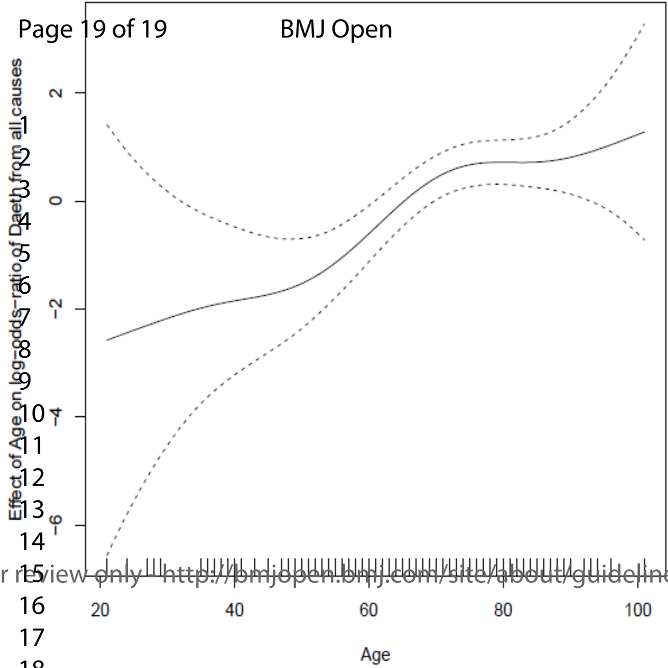

For peer review only - http://bmjopen.bmj.com/site/about/guidelines.xhtml

	Day 1	Day 2	Day 3	Day 4	Day 5
White Cell count x10^9/L	7·5 (4·2)	7·1 (4·3)	7·5 (4·3)	7·1 (4·7)	7·6 (4·6)
Lymphocytes x10^9/L	0·8 (0·6)	0·8 (0·5)	0·8 (0·5)	0·8 (0·6)	0·8 (0·5)
Neutrophils x10^9/L	6·1 (4·6)	5·7 (4·2)	5·6 (4·2)	5·6 (4·4)	6·4 (4·2)
Platelets x10^9/L	202 (121)	209 (125)	235 (131)	262 (161)	265 (164)
Bilirubin mg/dL	10 (8)	9 (7)	11 (7)	10(5)	11 (6)
Urea mmol/L	8 (6)	7(6)	7(6)	7(6)	8 (6)
Creatinine µmol/L	90 (56)	80(51)	74 (45)	75(40)	74 (52)
CRP mg/ml	98 (123)	115 (98)	122 (121)	121 (125)	117 (146)

Supplementary Table: Values of biomarkers at each day. Data is presented as median (interquartile range).

For peer review only

BMJ Open

The value of dynamic clinical and biomarker data for mortality risk prediction in COVID-19: A multi-centre retrospective cohort study

Journal:	*BMJ Open*
Manuscript ID	bmjopen-2020-041983.R1
Article Type:	Original research
Date Submitted by the Author:	03-Aug-2020
Complete List of Authors:	Berzuini, Carlo; University of Manchester, Biostatistics<br>Hannan, Cathal; Salford Royal Hospitals NHS Trust, Manchester Centre for Clinical Neurosciences<br>King, Andrew; Salford Royal Hospitals NHS Trust, Manchester Centre for Clinical Neurosciences<br>Vail, Andy; University of Manchester, Biostatistics<br>O'Leary, Claire; The University of Manchester, Division of Neuroscience and Experimental Psychology<br>Brough, David; The University of Manchester, Division of Neuroscience and Experimental Psychology<br>Galea, James; Cardiff and Vale University Health Board<br>Ogungbenro, Kayode; The University of Manchester, Pharmacy and Optometry<br>Wright, Megan; Salford Royal Hospitals NHS Trust, Manchester Centre for Clinical Neurosciences<br>Pathmanaban, Omar; Salford Royal Hospitals NHS Trust, Manchester Centre for Clinical Neurosciences<br>Hulme, Sharon; The University of Manchester, Division of Neuroscience and Experimental Psychology<br>Allan, Stuart; The University of Manchester, Division of Neuroscience and Experimental Psychology<br>Bernardinelli, Luisa; University of Pavia<br>Patel, Hiren; Salford Royal Hospitals NHS Trust, Manchester Centre for Clinical Neurosciences
**Primary Subject Heading**:	Research methods
Secondary Subject Heading:	Global health, Infectious diseases, Respiratory medicine
Keywords:	STATISTICS & RESEARCH METHODS, Respiratory infections < THORACIC MEDICINE, INTENSIVE & CRITICAL CARE

SCHOLARONE™
Manuscripts

I, the Submitting Author has the right to grant and does grant on behalf of all authors of the Work (as defined in the below author licence), an exclusive licence and/or a non-exclusive licence for contributions from authors who are: i) UK Crown employees; ii) where BMJ has agreed a CC-BY licence shall apply, and/or iii) in accordance with the terms applicable for US Federal Government officers or employees acting as part of their official duties; on a worldwide, perpetual, irrevocable, royalty-free basis to BMJ Publishing Group Ltd ("BMJ") its licensees and where the relevant Journal is co-owned by BMJ to the co-owners of the Journal, to publish the Work in this journal and any other BMJ products and to exploit all rights, as set out in our licence.

The Submitting Author accepts and understands that any supply made under these terms is made by BMJ to the Submitting Author unless you are acting as an employee on behalf of your employer or a postgraduate student of an affiliated institution which is paying any applicable article publishing charge ("APC") for Open Access articles. Where the Submitting Author wishes to make the Work available on an Open Access basis (and intends to pay the relevant APC), the terms of reuse of such Open Access shall be governed by a Creative Commons licence – details of these licences and which Creative Commons licence will apply to this Work are set out in our licence referred to above.

Other than as permitted in any relevant BMJ Author's Self Archiving Policies, I confirm this Work has not been accepted for publication elsewhere, is not being considered for publication elsewhere and does not duplicate material already published. I confirm all authors consent to publication of this Work and authorise the granting of this licence.

For peer review only

The value of dynamic clinical and biomarker data for mortality risk prediction in COVID-19: A multi-centre retrospective cohort study

Carlo Berzuini PhD, Cathal John Hannan MRCS, Andrew Thomas King FRCS(SN), Andy Vail MSc, Claire O'Leary PhD, David Brough PhD, James Galea PhD, Kayode Ogungbenro PhD, Megan Wright BSc, Omar Pathmanaban PhD, Sharon Hulme BSc, Stuart Allan PhD, Luisa Bernardinelli PhD, Hiren Patel PhD *.

Carlo Berzuini (carlo.berzuini@manchester.ac.uk). Professor of Biostatistics, Centre for Biostatistics, University of Manchester, Oxford Road, Manchester, M13 3PL, UK

Cathal John Hannan (cathalhannan@icloud.com). Neurosurgery Research Registrar, Manchester Centre for Clinical Neurosciences, Stott Lane, Manchester, M6 8HD, UK

Andrew Thomas King (Andrew.king@manchester.ac.uk). Professor of Neurosurgery, Manchester Centre for Clinical Neurosciences, Stott Lane, Manchester, M6 8HD, UK

Andy Vail (andy.vail@manchester.ac.uk). Professor of Biostatistics and Centre Lead, Centre for Biostatistics, University of Manchester, Oxford Road, Manchester, M13 3PL, UK

Claire O'Leary (Claire.oleary@manchester.ac.uk). Post-doctoral research associate, Division of Neuroscience and Experimental Psychology, University of Manchester, Oxford Road, Manchester, M13 3PL, UK

David Brough (David.brough@manchester.ac.uk). Professor of Neuroscience, Division of Neuroscience and Experimental Psychology, University of Manchester, Oxford Road, Manchester, M13 3PL, UK

James Galea (james.galea@manchester.ac.uk). Consultant Neurosurgeon, Cardiff and Vale University Health Board, Heath Park, Cardiff, CF14 4XW, UK

For peer review only

For peer review only - http://bmjopen.bmj.com/site/about/guidelines.xhtml

Kayode Ogungbenro (Kayode.Ogungbenro@manchester.ac.uk). Lecturer in Cancer Pharmocometrics, Division of Pharmacy and Optometry, University of Manchester, Oxford Road, Manchester, M13 3PL, UK

Megan Wright (megan.wright@manchester.ac.uk). Research Manager, Manchester Centre for Clinical Neurosciences, Stott Lane, Manchester, M6 8HD, UK

Omar Pathmanaban (omar.pathmanaban@manchester.ac.uk). Consultant Neurosurgeon, Manchester Centre for Clinical Neurosciences, Stott Lane, Manchester, M6 8HD, UK

Sharon Hulme (sharon.hulme@manchester.ac.uk). Research Manager, Division of Cardiovascular Sciences, University of Manchester, Oxford Road, Manchester, M13 3PL, UK

Stuart Allan (stuart.allan@manchester.ac.uk). Professor of Neuroscience, Division of Neuroscience and Experimental Psychology, University of Manchester, Oxford Road, Manchester, M13 3PL, UK

Luisa Bernardinelli (luiber04@unipv.it). Professor of Biostatistics, University of Pavia, Via Bassi, 21 - 27100 Pavia, Italy

Hiren Patel (hiren.patel@srft.nhs.uk). Consultant Neurosurgeon, Manchester Centre for Clinical Neurosciences, Stott Lane, Manchester, M6 8HD, UK

For peer review only

Contributor and Guarantor Information

All authors (CB, CH, AK, AV, CO, DB, JG, KO, MW, OP, SH, SA, LB, HP) were involved in the concept and design of the study which was led by HP and AV. HP, CH, MW acquired the data, and CB, LB, AV, HP, CH analysed and interpreted the data. CB, DB, SA, OP, JG, AV, CH, HP drafted the manuscript. All authors (CB, CH, AK, AV, CO, DB, JG, KO, MW, OP, SH, SA, LB, HP) were involved in revising the manuscript and contributed to the final draft. The corresponding author attests that all listed authors meet authorship criteria and that no others meeting the criteria have been omitted. HP acts as guarantor for the study.

Copyright

The Corresponding Author has the right to grant on behalf of all authors and does grant on behalf of all authors, a worldwide licence to the Publishers and its licensees in perpetuity, in all forms, formats and media (whether known now or created in the future), to i) publish, reproduce, distribute, display and store the Contribution, ii) translate the Contribution into other languages, create adaptations, reprints, include within collections and create summaries, extracts and/or, abstracts of the Contribution, iii) create any other derivative work(s) based on the Contribution, iv) to exploit all subsidiary rights in the Contribution, v) the inclusion of electronic links from the Contribution to third party material where-ever it may be located; and, vi) licence any third party to do any or all of the above."

Competing Interests Declaration

Swedish Orphan Biovitrum have provided investigational medicinal product for public-funded, peer-reviewed trials on which AK, AV, JG, HP, and SH are coinvestigators. The other authors declare no competing interests.

For peer review only

*Corresponding author: Manchester Centre for Clinical Neurosciences, Salford Royal NHS Foundation Trust, Manchester Academic Health Science Centre, Manchester, M6 8HD, UK.

Telephone:01612065059. Email: Hiren.Patel@srft.nhs.uk

Word Count 1768

Number of figures: 4

Number of tables: 2

Abstract

Objectives

Being able to predict which COVID-19 patients are going to deteriorate is important to help identify patients for clinical and research practice. Clinical prediction models play a critical role in this process, but current models are of limited value because they are typically restricted to baseline predictors and don't always use contemporary statistical methods. We sought to explore the benefits of incorporating dynamic changes in routinely measured biomarkers, non-linear effects and applying 'state of the art' statistical methods in the development of a prognostic model to predict death in hospitalised COVID-19 patients.

Design

Data were analysed from COVID-19 admissions to three hospital sites. Exploratory data analysis included a graphical approach to partial correlations. Dynamic biomarkers were considered up to five days following admission rather than depending solely on baseline or single time-point data. Marked departures from linear effects of covariates were identified by employing smoothing splines within a generalised additive modelling framework.

Setting

3 secondary and tertiary level centres in Greater Manchester, UK.

Participants

392 hospitalised patients with a diagnosis of COVID-19

Results

392 patients with a COVID-19 diagnosis were identified. Area under the receiver operating characteristic (ROC) curve increased from 0.73 using admission data alone to 0.75 when also considering results of baseline blood samples and to 0.83 when considering dynamic values of routinely collected markers. There was clear non-linearity in the association of age with patient outcome.

Conclusions

For peer review only

This study shows that clinical prediction models to predict death in hospitalised COVID-19 patients can be improved by taking into account both non-linear effects in covariates such as age and dynamic changes in values of biomarkers.

Strengths and Limitations.

- Incorporating routinely available blood tests performed over the first 5 days of hospital admission with clinical presentation data can enhance patient-level prediction of COIVD-19 progression.
- A larger dataset is needed to construct definitive prediction models.
- More sophisticated statistical exploitation of biomarker trajectories e. g using random effects models of biomarker evolution or 'conditional on outcome' models of biomarker evolution, could make clinical predictions models better still.

For peer review only

For peer review only - http://bmjopen.bmj.com/site/about/guidelines.xhtml

Introduction

Most patients with severe acute respiratory syndrome coronavirus 2 (SARS-Cov-2) experience mild symptoms. Some patients however experience significant symptoms requiring hospitalisation. The pandemic nature of the covid-19 outbreak has meant that hospital services and capacity can be overwhelmed.[1] A tool to predict which patients are likely to deteriorate or need intensive care would help clinicians, hospital managers and researchers make better decisions.

Several such models are reported for COVID-19 patients but have been criticised for risk of bias using the PROBAST (prediction model risk of bias assessment tool) criteria.[2] We have further concerns regarding the statistical tools used to develop models. First, current models typically only consider patient characteristics available at baseline and do not consider that COVID-19 patients' presentation and in hospital course is variable. Secondly, models routinely seek only linear effects of potential predictors on the outcome of interest although these are not always clinically plausible.

We sought here to explore the benefits of incorporating dynamic changes in routinely measured biomarkers, non-linear effects and applying 'state of the art' statistical methods in the development of a prognostic model to predict death in hospitalised COVID-19 patients.

Methods

Study Population

Admissions with confirmed COVID-19 (according to World Health Organisation guidance) at three hospitals in the Northern Care Alliance (Greater Manchester, UK) between 11th March and 17th April 2020 with a minimum of a three week follow-up were studied.[3]

Data Collection

Necessary approvals were obtained from the local Research and Innovation department. Research nurses abstracted data from the electronic patient records based on the International Severe Acute Respiratory and emerging Infection Consortium (ISARIC) data collection tool

For peer review only - http://bmjopen.bmj.com/site/about/guidelines.xhtml

but modified for use with this study.[4] The ISARIC study data were supplemented from electronic patient records with results of blood analyses performed as part of routine clinical care. The date of diagnosis was considered Day 1.

Data analysis

All data were subjected to range checks and validated for internal consistency and missing items then anonymised prior to transfer.

Selection of biomarkers

The initial list of potential markers was determined through review of the literature and availability within routinely collected data. Candidate variables were further screened using a graphical representation of the partial correlation structure stratified by survival status.[5] Routine bloods were typically analysed on alternate days. The assumption that the unrecorded values were missing at random was corroborated by inspection of joint bivariate plots of complete and incomplete observations made on each particular marker on consecutive days.[6] Then, each missing value of a marker was imputed by iterative sampling from its conditional predictive distribution given its past values, using R package MICE. [6 7]

Modelling

In this study we used the information contained in the clinical presentation data and available biomarkers (creatinine, lymphocyte count, etc.) to update, on a day-by-day basis, the patient's probability of death within 21 days

Initially, a binary logistic model for the all-cause mortality outcome using only clinical features at presentation was fitted initially. We then fitting separate logistic models for death for each day, using predictive variables identified from the partial correlation analysis described above. For each of the five days following hospital admission, we fitted a model based exclusively on data from subjects still alive at that day, with candidate predictors chosen out of the set of clinical variables and biomarker values collected until that day. This approach meant that for each of the first five days following admission, a sequence of day specific mortality prediction models were available. We subsequently fitted each model within a generalised additive modelling (GAM) framework involving smoothing splines to detect marked departures from linearity for continuous predictors and undertook data transformations (e.g. log transformation of concentrations) as indicated.[8] A standard logistic

version of the model was then fitted. We used the Akaike Information Criterion (AIC) to choose between logistic models and assessed predictive performance using the area under a ten-fold, cross-validated Receiver Operating Characteristic (ROC) curve.

Patient and Public involvement.

There was no involvement of patients or the general public in the design or delivery of this study. This was because of the acute nature and fast moving pace of the disease studies and because access to patients and the public was limited at this time.

Results

A total of 392 patients with a COVID-19 diagnosis were admitted during the study period. Table 1 provides a summary of their demographic and clinical features including medical history. Blood samples were typically requested every other day following admission (supplementary Table).

For an informal analysis of biomarker relationships we analysed partial correlations between seven potential inflammatory markers in each of the two survival groups (Fig 1). Amongst survivors, the anticipated correlations between lymphocytes (Lym) and neutrophils (Neu, via white cell count) and between creatinine (Cre) and urea (Ure) were present, but these correlations were found to be significantly lower among the decedents, suggesting the possible presence of differences in the neutrophil/lymphocyte and urea/creatinine ratios between the two outcome groups.

Inclusion of admission biomarker data did not improve the predictive value of the model over clinical data alone. Incorporation of post admission dynamic biomarker data did however increase the discriminative ability of the model (Fig 2). Estimates from the best fitting model at Day 5 (Table 2) show strongly statistically significant term(s) reflecting post-baseline biomarker changes that that can be readily visualised (Fig 3). In addition to age and disease severity, the most recent neutrophil:lymphocyte ratio and the two most recent (and therefore recent change in) urea:creatinine ratios were generally predictive. There was a marked non-linearity in the effect of age (Fig 4).

Discussion

These results suggest that using dynamic data is better than using baseline initial presentation data to predict death in COVID-19 patients. Even with a local dataset of just 392 COVID-19

For peer review only

For peer review only - http://bmjopen.bmj.com/site/about/guidelines.xhtml

admissions we were able to identify clear benefit from exploiting dynamic biomarker data and marked non-linearity in the effects of commonly used factors to predict outcomes. Our findings should be taken as indicative of the benefits of 'state of the art' statistical methodology but also the necessary collaboration between statisticians and clinicians as this statistical methodology is not readily accessible to most researchers. Identification and validation of anything approaching a definitive predictive model would require substantially larger sample sizes.[9]

Neither the non-linear effect of age after allowance for other factors nor the particular biomarkers identified within this dataset are surprising. Others have also observed associations of mortality with age, and clinical and biochemical markers of disease severity (e.g. neutrophil/lymphocyte ratio).[10-12] There have been few studies investigating dynamic changes in patient biomarkers for mortality prediction in COVID-19; one such study of 548 patients in China also demonstrated that the neutrophil:lymphocyte ratio in survivors and non-survivors became increasingly divergent throughout their hospital admission.[13] Chen et al derived their prognostic score from an analysis based on a Cox's regression model with their candidate predictive variables taken at baseline. They incorporated in their analysis the slope of a line fitted to the first and last measurements of each particular marker to model changes over time. Chen et al approach has advantages and disadvantages. Their model captures duration information but does not involve choice of time horizon for prediction. Their predictions are arguably limited because they are not updated daily and depend on the assumption that marker evolution is linear and summarised by a straight line between initial and final values.

A smaller study limited to patients with severe COVID-19 also revealed a progressive increase in neutrophil count and plasma interleukin-6 concentration in the decedents when compared to the survivors, but the authors did not perform any assessment of the predictive value associated with dynamic changes in these laboratory parameters.[14]

Similarly, renal injury has also been shown to be common in COVID-19 patients and is associated with a worse outcome.[15] The reason for this is not clear. There is emerging evidence that SARS-CoV-2 infection can directly harm the kidneys. The worsening urea/creatinine ratio observed in our data set may also reflect either the therapeutic effects of fluid restriction to treat severe ARDS or evidence of multiorgan dysfunction.[11 16] Regardless of the cause, the impact of the urea/creatinine level on death was not evident at presentation but became a significant predictor of death in our model over time. This observation illustrates the benefit of

For peer review only - http://bmjopen.bmj.com/site/about/guidelines.xhtml

taking into account improvements and deterioration in daily blood test results as well as initial presentation factors when calculating the probability of death. Improving the accuracy of prediction models using this approach is likely to be successful in informing clinical decision making, resource planning and communication with patients and relatives.

We acknowledge that our study has some limitations. We would have liked to consider other outcomes including dynamic changes in clinical variables, as well as disease end points such as the incidence of ARDS and ICU admission. Dynamic clinical data were not included because it was less reliable to obtain compared to blood biomarker data, and a consistent diagnosis of ARDS, and dates of onset or admission to ICU were also not routinely available. Although we have undertaken internal cross-validation to ensure unbiased comparison of ROC curves we have not considered calibration. We do not wish to make any claim for the value of our current models at each day based on the small sample size available to us locally. With only three hospital sites contributing during the first wave, and because of significant time/resource pressures during the pandemic we did not have sufficient data to construct definitive prediction models or to follow-up patients beyond 3 weeks. More sophisticated exploitation of biomarker trajectories through, for example, approaches based on random effects models of biomarker evolution or ‘conditional on outcome’ models of biomarker evolution, would also require more data and be expected to add further insights.[17-19]

Clinical prediction models are important and can help in clinical decision making, resource allocation and optimal selection of trial participants for investigational treatments. In the setting of an infectious disease pandemic- affecting all geographic and socioeconomic groups – using routinely available blood tests to inform prediction models has obvious advantages over less widely available, but perhaps more specific, biomarkers of disease severity. Until investigators incorporate such data in participant selection it is unlikely that future trials will be able to accurately target those patients most likely to benefit from therapies such as immunomodulation. Overall benefits will be ‘diluted’ and potentially reversed by inclusion of participants who have nothing to gain and, in theory, may be harmed by restriction of a healthy inflammatory response.[20] The consequence of poorly considered eligibility criteria may therefore be to erroneously dismiss therapies that could benefit those at highest risk from COVID-19.

Data sharing statement.

Our data would be made available to reasonable requests.

For peer review only - http://bmjopen.bmj.com/site/about/guidelines.xhtml

References

1. Emanuel EJ, Persad G, Upshur R, et al. Fair Allocation of Scarce Medical Resources in the Time of Covid-19. *New England Journal of Medicine* 2020;382(21):2049-55. doi: 10.1056/NEJMsb2005114
2. Wynants L, Van Calster B, Collins GS, et al. Prediction models for diagnosis and prognosis of covid-19: systematic review and critical appraisal. *BMJ* 2020;369:m1328. doi: 10.1136/bmj.m1328
3. WHO. Laboratory testing for coronavirus disease

(COVID-19) in suspected human cases. 2020
4. International Severe Acute Respiratory and emerging Infection Consortium. URL: https://isaric.tghn.org Accessed 28/05/2020.
5. Friedman J, Hastie T, Tibshirani R. Sparse inverse covariance estimation with the graphical lasso. *Biostatistics* 2007;9(3):432-41. doi: 10.1093/biostatistics/kxm045
6. RUBIN DB. Inference and missing data. *Biometrika* 1976;63(3):581-92. doi: 10.1093/biomet/63.3.581
7. Azur MJ, Stuart EA, Frangakis C, et al. Multiple imputation by chained equations: what is it and how does it work? *Int J Methods Psychiatr Res* 2011;20(1):40-9. doi: 10.1002/mpr.329 [published Online First: 2011/04/19]
8. Hastie T, Tibshirani R. Generalized additive models. 2017
9. Wynants L, Van Calster B, Bonten MMJ, et al. Prediction models for diagnosis and prognosis of covid-19 infection: systematic review and critical appraisal. *BMJ* 2020;369:m1328. doi: 10.1136/bmj.m1328
10. Huang C, Wang Y, Li X, et al. Clinical features of patients infected with 2019 novel coronavirus in Wuhan, China. *The Lancet* 2020;395(10223):497-506. doi: 10.1016/S0140-6736(20)30183-5
11. Zhou F, Yu T, Du R, et al. Clinical course and risk factors for mortality of adult inpatients with COVID-19 in Wuhan, China: a retrospective cohort study. *The Lancet* 2020;395(10229):1054-62. doi: 10.1016/S0140-6736(20)30566-3
12. Ruan Q, Yang K, Wang W, et al. Clinical predictors of mortality due to COVID-19 based on an analysis of data of 150 patients from Wuhan, China. *Intensive Care Medicine* 2020;46(5):846-48. doi: 10.1007/s00134-020-05991-x
13. Chen R, Sang L, Jiang M, et al. Longitudinal hematologic and immunologic variations associated with the progression of COVID-19 patients in China. *J Allergy Clin Immunol* 2020;146(1):89-100. doi: 10.1016/j.jaci.2020.05.003 [published Online First: 2020/05/11]
14. Pan F, Yang L, Li Y, et al. Factors associated with death outcome in patients with severe coronavirus disease-19 (COVID-19): a case-control study. *Int J Med Sci* 2020;17(9):1281-92. doi: 10.7150/ijms.46614
15. Cheng Y, Luo R, Wang K, et al. Kidney disease is associated with in-hospital death of patients with COVID-19. *Kidney Int* 2020;97(5):829-38. doi: 10.1016/j.kint.2020.03.005 [published Online First: 2020/04/06]
16. Su H, Yang M, Wan C, et al. Renal histopathological analysis of 26 postmortem findings of patients with COVID-19 in China. *Kidney International* doi: 10.1016/j.kint.2020.04.003
17. Berzuini C, Larizza C. A unified approach for modeling longitudinal and failure time data, with application in medical monitoring. *IEEE Transactions on Pattern Analysis and Machine Intelligence* 1996;18(2):109-23.
18. Albert PS. A linear mixed model for predicting a binary event from longitudinal data under random effects misspecification. *Stat Med* 2012;31(2):143-54. doi: 10.1002/sim.4405
19. Liu D, Albert PS. Combination of longitudinal biomarkers in predicting binary events. *Biostatistics* 2014;15(4):706-18. doi: 10.1093/biostatistics/kxu020 [published Online First: 2014/05/17]
20. King A, Vail A, O'Leary C, et al. Anakinra in COVID-19: important considerations for clinical trials. *The Lancet Rheumatology* doi: 10.1016/S2665-9913(20)30160-0

For peer review only - http://bmjopen.bmj.com/site/about/guidelines.xhtml

Acknowledgments

The Northern Care Alliance research delivery team who collected the data for the patients, and the Salford Royal business analysis team who helped in providing access to the daily blood and clinical observation results. This report was also made possible by the provision of the data collection tool through the efforts and expertise of the ISARIC Team (www.isaric.tghn.org).

Role of funding source

AK, AV, JG, HP, and SH are funded by the National Institute for Health Research Efficacy and Mechanism Evaluation Programme Ref: 14/209/07. DB is funded by MRC grant MR/T016515/1.

For peer review only

Figure Legends:

Figure 1. Partial correlations between biomarkers. Nodes represent average marker levels day 2 to 5 and edges represent partial correlations, as calculated from the survivors (left) and from the decedents (right). Broader lines indicate stronger relationships. Blr Bilirubin, CRP- C-Reactive Protein, Crt-Creatinine, Lym-Lymphocytes, Ntr-Neutrophils, WCC- White Cell Count, Ure-Urea.

Figure 2. ROC curves for three models: solid line indicates model considering only clinical factors at baseline (Area under ROC curve = 0.73); finely dotted line indicates model extended to consider also biomarker data from baseline sample (Area under ROC curve = 0.75); top line indicates model at 5 days extended to consider dynamic changes in biomarker data (Area under ROC curve = 0.83). Note that models are not nested

Figure 3. Violin plots showing distribution at each day of admission, stratified by survival status, for biomarkers identified by statistical modelling. Panel A: log transformed neutrophil ($x10^{\wedge 9}$/L):lymphocyte ($x10^{\wedge 9}$/L) ratio. Panel B: log transformed urea (mmol/L):creatinine (μmol/L) ratio. Survivors (white) on left and decedents (shaded) on right.

Figure 4. Spline plot demonstrating marked non-linearity in relationship between age and outcome after adjustment for other factors included in the final model.

For peer review only

Table 1. Demographic, clinical and medical history factors considered at baseline.

	Overall dataset
Number of patients	392
Age, median (IQR)	71(22)
Gender Male: Female ratio	65:35
Median time to hospitalisation following disease onset (IQR) days	5(8)
Initial symptoms (%)	
Fever	223 (57%)
Cough	240(61%)
Dyspnoea	245 (65%)
Fatigue	127 (37%)
Muscle ache	53 (16%)
Co-morbidities	
Cardiovascular disease	108 (28%)
Chronic Respiratory disease (inc asthma)	110 (28%)
Chronic Renal disease	45 (12%)
Chronic Liver disease	14 (2%)
Obesity	34 (10%)
Diabetes	95 (24%)
Dementia	49 (13%)
Current smoker	24 (7%)
Presenting clinical features	
Requirement for supplemental O_2	125(37%)
Oxygen Saturation < 90	59 (17%)
Respiratory rate>24	109 (30%)
Temperature ≥38°C	168 (45%)
MAP≤70mmHg	30 (8%)
Outcomes	
Acute Respiratory Distress Syndrome	47 (17%)
Non-Invasive Ventilation	25 (9%)
Need for ICU care	31 (12%)
Invasive Ventilation	14 (5%)
Death	110 (27%)

For peer review only

Table 2. Estimated coefficients (Est) with their standard error (se) and p-value. Note that different variables are selected at different days so that models are not nested. Neut/Lymp- Neurotrophil/Lymphocyte, Creat- Creatinine, D- Day, O_{2-} Oxygen. * The addition of biomarker data on day 2 did not contribute any additional predictive power of that obtained at Day 1.

	**Clinical data alone Day 1**			**Clinical data + Day 1* biomarker data**			**Day 3**			**Day 4**			**Day 5**		
**Predictor**	**Est**	**se**	***p***	**Est**	**se**	***p***	**Est**	**se**	***p***	**Est**	**se**	***p***	**Est**	**se**	***p***
**Intercept**	0·31	1·36	0·8	-4·36	1·35	0·001	0·67	1·97	0·73	0·005	1·86	0·99	-0·20	1·68	0·9
**log Neut/Lymp D1**				0·28	0·16	0·08									
**log Neut/Lymp D3**							0·41	0·19	0·03						
**log Neut/Lymp D4**										0·48	0·2	0·02			
**log Neut/Lymp D5**													0·52	0·21	0·01
**log Urea/Creat D2**							-4·22	1·24	0·0007						
**log Urea/Creat D3**							5·13	1·30	0·0001						
**log Urea/Creat D4**										1·08	0·35	0·002	-4·97	1·72	0·0003
**log Urea/Creat D5**													6·32	1·77	0·0004
**Age (Years)**	0·13	0·026	<0·0001	0·073	0·012	<0·0001	0·069	0·013	<0·0001	0·071	0·012	<0·0001	0·066	0·012	<0·0001
**O_2 Saturation**	-0·03	0·013	0·05	-0·03	0·012	0·01	-0·03	0·013	0·03	-0·03	0·012	0·02			
**Respiratory Rate**	0·05	0·022	0·02	0·085	0·022	0·0001	0·08	0·023	0·0003	0·087	0·022	0·0001	0·09	0·022	0·0001
**Smoking**	0·44	0·29	0·1	0·7	0·267	0·01	0·8	0·27	0·004	0·71	0·27	0·008	0·76	0·28	0·006

For peer review only

For peer review only - http://bmjopen.bmj.com/site/about/guidelines.xhtml

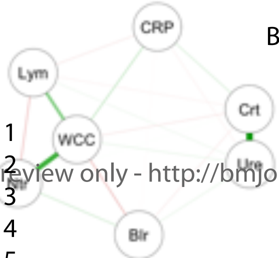

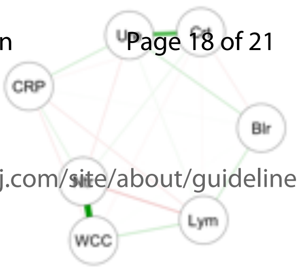

er review only - http://bmjopen.bmj.com/site/about/guideline

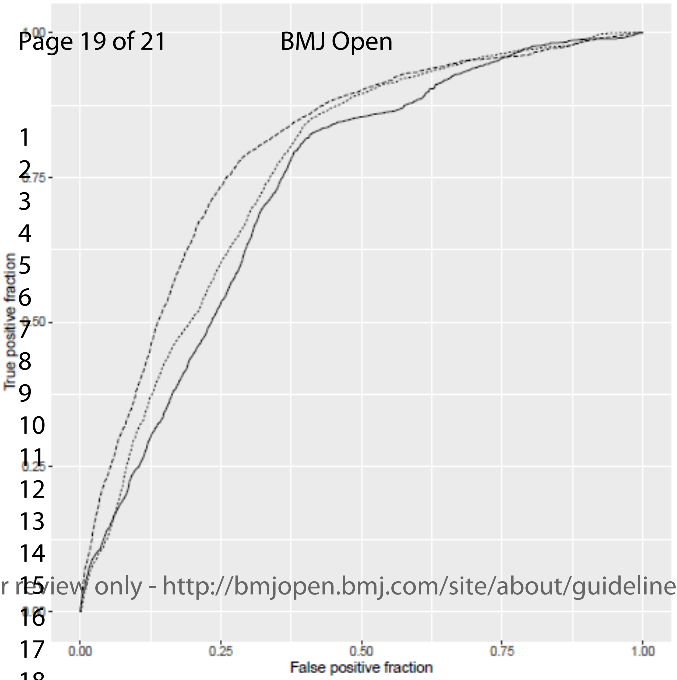

er review only - http://bmjopen.bmj.com/site/about/guideline

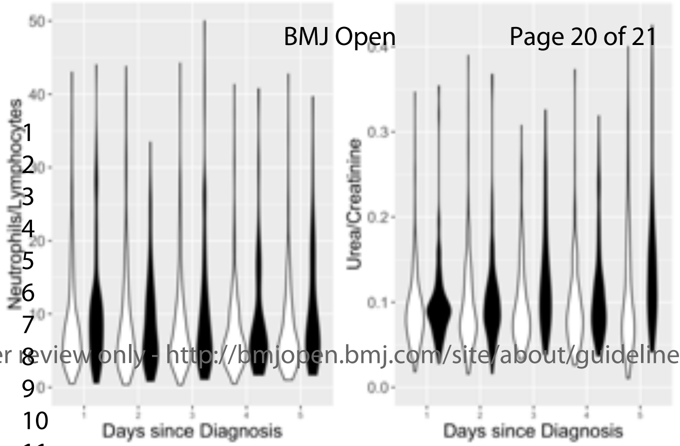

er review only - http://bmjopen.bmj.com/site/about/guideline

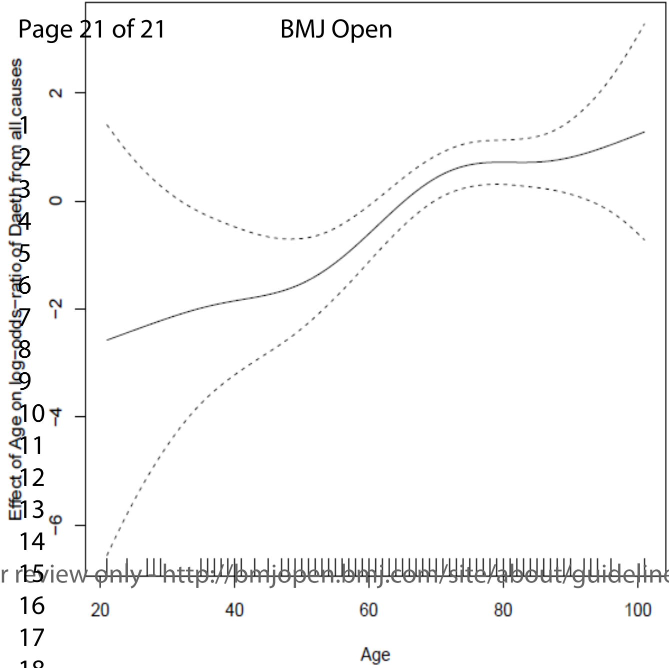

er review only - http://bmjopen.bmj.com/site/about/guideline

	Day 1	Day 2	Day 3	Day 4	Day 5
White Cell count x10^9/L	7·5 (4·2)	7·1 (4·3)	7·5 (4·3)	7·1 (4·7)	7·6 (4·6)
Lymphocytes x10^9/L	0·8 (0·6)	0·8 (0·5)	0·8 (0·5)	0·8 (0·6)	0·8 (0·5)
Neutrophils x10^9/L	6·1 (4·6)	5·7 (4·2)	5·6 (4·2)	5·6 (4·4)	6·4 (4·2)
Platelets x10^9/L	202 (121)	209 (125)	235 (131)	262 (161)	265 (164)
Bilirubin mg/dL	10 (8)	9 (7)	11 (7)	10(5)	11 (6)
Urea mmol/L	8 (6)	7(6)	7(6)	7(6)	8 (6)
Creatinine µmol/L	90 (56)	80(51)	74 (45)	75(40)	74 (52)
CRP mg/ml	98 (123)	115 (98)	122 (121)	121 (125)	117 (146)

Supplementary Table: Values of biomarkers at each day. Data is presented as median (interquartile range).

For peer review only

TRIPOD Checklist: Prediction Model Development

Section/Topic	Item	Checklist Item	Page
**Title and abstract**			
Title	1	Identify the study as developing and/or validating a multivariable prediction model, the target population, and the outcome to be predicted.	1
Abstract	2	Provide a summary of objectives, study design, setting, participants, sample size, predictors, outcome, statistical analysis, results, and conclusions.	4
**Introduction**			
Background and objectives	3a	Explain the medical context (including whether diagnostic or prognostic) and rationale for developing or validating the multivariable prediction model, including references to existing models.	6
	3b	Specify the objectives, including whether the study describes the development or validation of the model or both.	7
**Methods**			
Source of data	4a	Describe the study design or source of data (e.g., randomized trial, cohort, or registry data), separately for the development and validation data sets, if applicable.	7
	4b	Specify the key study dates, including start of accrual; end of accrual; and, if applicable, end of follow-up.	7
Participants	5a	Specify key elements of the study setting (e.g., primary care, secondary care, general population) including number and location of centres.	6
	5b	Describe eligibility criteria for participants.	6
	5c	Give details of treatments received, if relevant.	n/a
Outcome	6a	Clearly define the outcome that is predicted by the prediction model, including how and when assessed.	6,7
	6b	Report any actions to blind assessment of the outcome to be predicted.	n/a
Predictors	7a	Clearly define all predictors used in developing or validating the multivariable prediction model, including how and when they were measured.	7
	7b	Report any actions to blind assessment of predictors for the outcome and other predictors.	n/a
Sample size	8	Explain how the study size was arrived at.	n/a
Missing data	9	Describe how missing data were handled (e.g., complete-case analysis, single imputation, multiple imputation) with details of any imputation method.	7
Statistical analysis methods	10a	Describe how predictors were handled in the analyses.	7
	10b	Specify type of model, all model-building procedures (including any predictor selection), and method for internal validation.	7
	10d	Specify all measures used to assess model performance and, if relevant, to compare multiple models.	7
Risk groups	11	Provide details on how risk groups were created, if done.	n/a
**Results**			
Participants	13a	Describe the flow of participants through the study, including the number of participants with and without the outcome and, if applicable, a summary of the follow-up time. A diagram may be helpful.	13
	13b	Describe the characteristics of the participants (basic demographics, clinical features, available predictors), including the number of participants with missing data for predictors and outcome.	13
Model development	14a	Specify the number of participants and outcome events in each analysis.	13
	14b	If done, report the unadjusted association between each candidate predictor and outcome.	n/a
Model specification	15a	Present the full prediction model to allow predictions for individuals (i.e., all regression coefficients, and model intercept or baseline survival at a given time point).	14
	15b	Explain how to the use the prediction model.	8
Model performance	16	Report performance measures (with CIs) for the prediction model.	Figure 3
**Discussion**			
Limitations	18	Discuss any limitations of the study (such as nonrepresentative sample, few events per predictor, missing data).	9
Interpretation	19b	Give an overall interpretation of the results, considering objectives, limitations, and results from similar studies, and other relevant evidence.	8
Implications	20	Discuss the potential clinical use of the model and implications for future research.	9
**Other information**			
Supplementary information	21	Provide information about the availability of supplementary resources, such as study protocol, Web calculator, and data sets.	10
Funding	22	Give the source of funding and the role of the funders for the present study.	11

We recommend using the TRIPOD Checklist in conjunction with the TRIPOD Explanation and Elaboration document.
